# Value chain carbon footprints of Chinese listed companies

**DOI:** 10.1038/s41467-023-38479-5

**Published:** 2023-05-16

**Authors:** Zengkai Zhang, Jiaoyan Li, Dabo Guan

**Affiliations:** 1 State Key Laboratory of Marine Environmental Science, College of the Environment and Ecology, Xiamen University, 361102 Xiamen, Fujian China; 2 College of Management and Economics, Tianjin University, 300072 Tianjin, China; 3 Department of Earth System Science, Tsinghua University, 100084 Beijing, China; 4 The Bartlett School of Construction and Project Management, University College London, London, WC1E 7HB UK

**Keywords:** Climate-change mitigation, Climate-change policy

## Abstract

Measuring the value chain carbon footprints of listed companies is essential for cumulative climate actions and climate-efficient capital allocation. We trace the carbon emissions embodied in the value chains of Chinese listed companies and find that there is an increasing trend in terms of the carbon footprints of listed companies over the period 2010–2019. In 2019, the direct emissions from these companies reached 1.9 billion tonnes, accounting for 18.3% of national emissions. The indirect emissions were well over twice as large as the direct emissions from 2010 to 2019. Energy, construction and finance companies tend to have a greater volume of value chain carbon footprints, yet the distribution of their carbon footprints varies significantly. Finally, we apply the results to evaluate the financed emissions of leading asset managers’ equity portfolio investment in China’s stock market.

## Abstract (web summary)

A paper led by Prof. Zhang evaluates the value chain carbon footprints of Chinese listed companies. The results could encourage collaborative climate actions along value chains and help investors understand the environmental impacts of their investment.

## Introduction

In 2021, Generation Investment Management, an investment management firm, reported that publicly traded companies are responsible for 40% of global greenhouse gas emissions and that cutting value chain carbon footprints should be a priority^1^. The volume of indirect emissions that occur in the value chain (Scope 3 carbon footprint) of listed companies tends to be significantly greater than their operational emissions (Scope 1 carbon footprint) and emissions from the generation of purchased electricity, steam, heating or cooling (Scope 2 carbon footprint)^2^. However, it is difficult for listed companies to obtain emissions data from their upstream suppliers and downstream buyers to measure their value chain carbon footprint. The problem of information on value chain emissions being missing or inaccurate^3^ is extremely serious. According to the Carbon Disclosure Project (CDP), which invites companies to disclose climate change data through questionnaires voluntarily and provides it to the marketplace, in 2021, only 82 listed companies(1.7% of Chinese listed companies) in China provided comprehensive reports on climate change and environmental issues^4^. The present study attempts to provide a comprehensive evaluation of the value chain carbon footprints of all Chinese listed companies.

China has pledged to peak its carbon emissions by 2030 and reach carbon neutrality by 2060^5^. Companies—particularly publicly traded companies—are under increasing pressure to cut their carbon footprints, as these companies usually play key roles in inspiring collaborative climate actions along value chains. Carbon footprint measurement is the basis for low-carbon management practices^6^, such as the identification of mitigation hotspots and the adoption of more targeted climate actions. In addition, investors, who can influence the operation of listed companies through the stock market, are paying increasing attention to the environmental impacts associated with their investment profiles. As climate regulations become increasingly stringent, investors allocate a greater share of their capital to the stocks of listed companies with lower carbon footprints to reduce the climate risk associated with their investment. Information on the value chain carbon footprints of listed companies is also the basis for climate-efficient capital allocation. The results of the present study could help both investors and business managers gain a better understanding of the carbon footprints of listed companies.

The Greenhouse Gas Protocol provides the most widely used carbon accounting standards and recommends a list of techniques and databases with which companies can measure their value chain carbon footprints^7^. The economic input‒output life-cycle assessment (EIO-LCA) method is a suggested approach to tracing carbon footprints along the value chain. The traditional EIO-LCA method is based on the Leontief input‒output (IO) framework^8^, which is suitable for evaluating the cradle-to-gate environmental impacts associated with final demand. However, a company’s output is not always delivered to final consumers, and most of the outputs are further processed by other businesses. Therefore, the traditional EIO-LCA method may underestimate the carbon emissions embodied in the downstream value chains of a company^9^. To fill this gap, we integrate the hypothetical extraction method (HEM) with a unified IO framework^10–14^, which captures the pre- and postproduction stages of output. Then, we estimate the carbon footprints of listed companies based on the output listed in their annual reports. Another advantage of this method is that it integrates structure path analysis, allowing us to trace the carbon emissions embodied in different tiers of the value chain. Here, we apply the proposed method to calculate the carbon footprints of listed companies’ domestic operations in China over the period 2010–2019.

## Results

### Carbon footprints of listed companies from 2010 to 2019

**Fig. 1:**
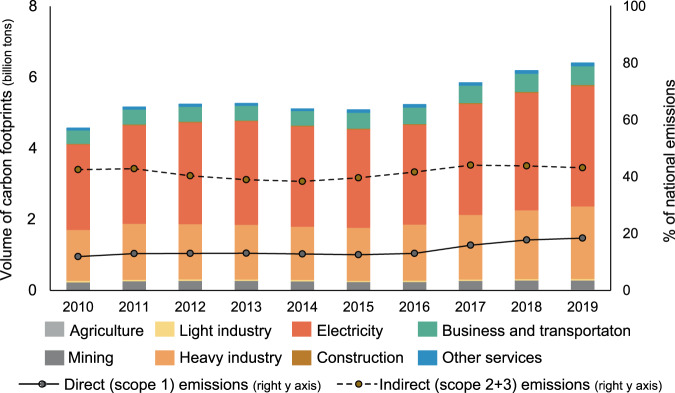
Carbon footprints of Chinese listed companies. The carbon footprints of Chinese listed companies are classified into eight sectors, which are symbolized by different colors. We obtained the volume of direct emissions and indirect emissions (Supplementary Information 1.4 and 2.3), which contain the Scope 2 and Scope 3 carbon footprints. The solid line presents the ratio of direct emissions to national emissions, and the dotted lines represent the ratio of indirect emissions to national emissions.

According to the operating information of all the listed companies (~3800 in total) obtained from their annual reports that are issued obligatorily, in 2019, the gross revenue of all the Chinese listed companies reached 49.7 trillion yuan, accounting for approximately 18.7% of gross output in 2019. The direct emissions from these companies reached 1.9 billion tonnes (Supplementary Information 1.4), accounting for 18.3% of national emissions. The indirect emissions are well over twice as large as the direct emissions from 2010 to 2019, which indicates the huge potential for China to scale up climate action by mobilizing the value chain emission reduction of its listed companies^15^. Figure 1 further presents the changing trends in the carbon footprints of Chinese listed companies over the period 2010–2019.

Over the period 2010–2013, the volume of China’s gross CO_2_ emissions from fossil fuels increased rapidly and peaked in 2013. The carbon footprints of listed companies also increased quickly, peaking at 5.3 billion tonnes in 2013. After 2013, there was a slight decrease in national CO_2_ emissions^16^, and the total carbon footprints of listed companies decreased in 2014 and 2015 as well. In 2015, the stock market crash in China reduced the productive activities of Chinese listed companies^17^, and thus, the total carbon footprint of listed companies decreased to 5.1 billion tonnes in 2015. After 2015, China’s CO_2_ emissions from fossil fuels increased again, as did the carbon footprints of listed companies. In 2019, the volume of the carbon footprints of listed companies reached a second peak of 6.4 billion tonnes. Over the period 2010–2019, there was an increasing trend in the volume of emissions in which listed companies could have a direct influence (Supplementary Information 1.4). The share of direct emissions of listed companies increased from 11.8% in 2010 to 18.3% in 2019. Listed companies could adopt more straightforward measures, such as improving production technologies to combust less fossil fuel, to mitigate these emissions. In 2010, 42.4% of national emissions were indirectly emitted due to the production activities of Chinese listed companies, and this share was 43.0% in 2019. Listed companies can collaborate with their value chain partners and other industry leaders, who are often publicly listed, and have a strong influence over the whole value chain.

The carbon footprints of listed companies originate from eight sectors (Fig. 1). The electricity generation sector by far corresponds to the greatest share of those emissions. This is mainly due to China’s high-carbon-intensive electricity generation mix^18^. Heavy industry accounted for the second-greatest share of the carbon footprint of listed companies over the study period. In 2019, more than half (52.9%) of the carbon footprint was from the electricity generation sector, and the share was 32.0% for the heavy industry sector. Climate actions concerning the emissions of the electricity generation and heavy industry sectors, such as switching to greener electricity, are essential to reducing the carbon footprints of listed companies. For instance, Apple is cooperating with its manufacturing partners to reduce its carbon footprint, and its manufacturing partners need to use 100% renewable energy for production^19^. The business and transportation sector accounts for approximately 8.0% of the carbon footprints of Chinese listed companies, and the share has remained relatively stable over the study period. Controlling transportation-related emissions is also necessary for mitigating the carbon footprints of listed companies. The mining sector accounts for approximately 4.0% of the carbon footprints of Chinese listed companies. Although the volume of direct emissions in the mining sector is relatively small, mining companies provide the necessary materials to support downstream users. Therefore, listed companies could cooperate with not only their upstream suppliers but also their downstream users. The share of the other four sectors is only approximately 3.0%. For example, the share of the construction sector is only 0.5%, mainly due to its low direct carbon intensity^20^. We further provide a more detailed analysis of value chain carbon footprints at the company level.

### Top listed companies by volume of value chain carbon footprints

**Fig. 2:**
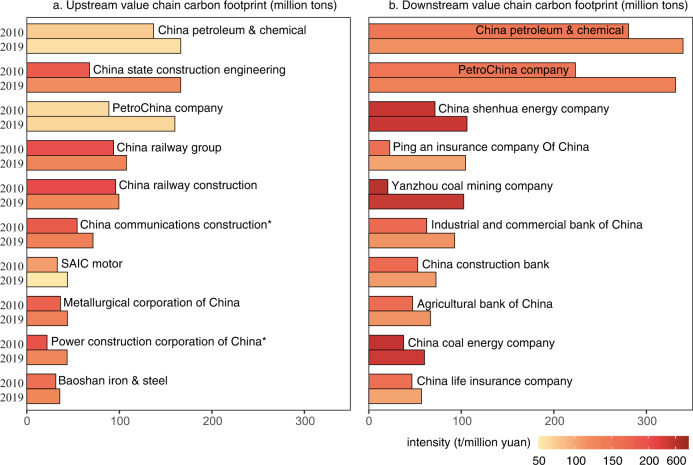
Value chain carbon footprints of the top 10 listed companies by volume (million tons) in 2010 and 2019. Panel **a** shows the top 10 listed companies that are determined by the volume of upstream value chain carbon footprints in 2019. Since China Communications Construction and the Power Construction Corporation of China had not been listed in the Chinese stock market in 2010 (with superscript *), we replaced the data in 2010 with the data from 2012 and 2011, respectively, which are the years these companies became listed companies. Panel **b** shows the top 10 listed companies that are determined by the volume of downstream value chain carbon footprints in 2019. The color of each bar represents the carbon footprint intensity (volume of the carbon footprint divided by the revenue).

In 2019, the volume of value chain carbon footprints of approximately 80% of listed companies was three times greater than direct emissions. There is enormous potential for listed companies to implement collaborative climate actions with their upstream suppliers and downstream users. We calculate the volume of value chain carbon footprints, which are further divided into upstream and downstream parts, of Chinese listed companies over the period 2010–2019. Figure 2 presents the results of the top 10 listed companies by volume of upstream and downstream value chain carbon footprints.

In 2019, China Petroleum & Chemical Corporation had the largest upstream value chain carbon footprint (166.4 Mt CO_2_) among all Chinese listed companies (~3800 in total), followed by China State Construction Engineering (166.2 Mt CO_2_) and PetroChina Company (160.0 Mt CO_2_). The production activities of petroleum chemical and construction companies heavily rely on upstream raw materials, such as crude oil and construction materials. In addition, other manufacturing companies, such as Metallurgical Corporation of China and SAIC Motor, also correspond to a relatively large volume of upstream value chain carbon footprints. It is necessary for these companies, especially for construction enterprises, which have greater upstream value chain carbon footprint intensity, to cooperate with their upstream suppliers to make their supply chain cleaner. Over the study period 2010–2019, the volume of upstream value chain carbon footprints of these ten listed companies increased, yet there was a decreasing trend in value chain carbon footprint intensity. For instance, the upstream value chain carbon footprint intensity of China State Construction Engineering decreased from 182.9 t/million yuan in 2010 to 117.1 t/million yuan in 2019.

Listed companies that are in upstream positions of production networks and they supply energy or capital to support the production activities of downstream producers tend to have greater downstream value chain carbon footprints than other companies (Supplementary Information 1.3). For instance, the investment category, which includes emissions financed by loans and investments of large financial institutions^21^, is included in the seven categories of downstream footprints of GHG accounting^2^; therefore, financial institutions often hold a large amount of downstream carbon footprints^22, 23^. The top ten companies by volume of downstream value chain carbon footprints are energy companies, such as PetroChina, and finance companies, such as the Industrial and Commercial Bank, the volume of downstream value chain carbon footprints of which reached 331.5 and 92.7 Mt CO_2_ in 2019, respectively. Therefore, it is necessary to promote the development of technology in terms of the clean utilization of fossil fuel resources and encourage financial flows toward greener economic activities. For instance, coal mining companies, which have large downstream value chain carbon footprint intensity, could develop coal washing and processing capacities to raise the efficiency of combustion and reduce the carbon emissions enabled by their extraction activities^24^. Energy companies, as well as financial companies, are playing an increasingly important role in climate change actions. In 2020, China’s five ministries issued the “Guidance on Promoting Investment and Financing to Address Climate Change”, which states that finance enterprises must reduce the loans issued to traditional thermal power companies. Traditional energy-intensive companies would have to reduce their carbon intensity to attract financial support. Over the study period, there was a decreasing trend in downstream value chain carbon footprint intensity.

### Distribution of carbon footprints along the value chain

**Fig. 3:**
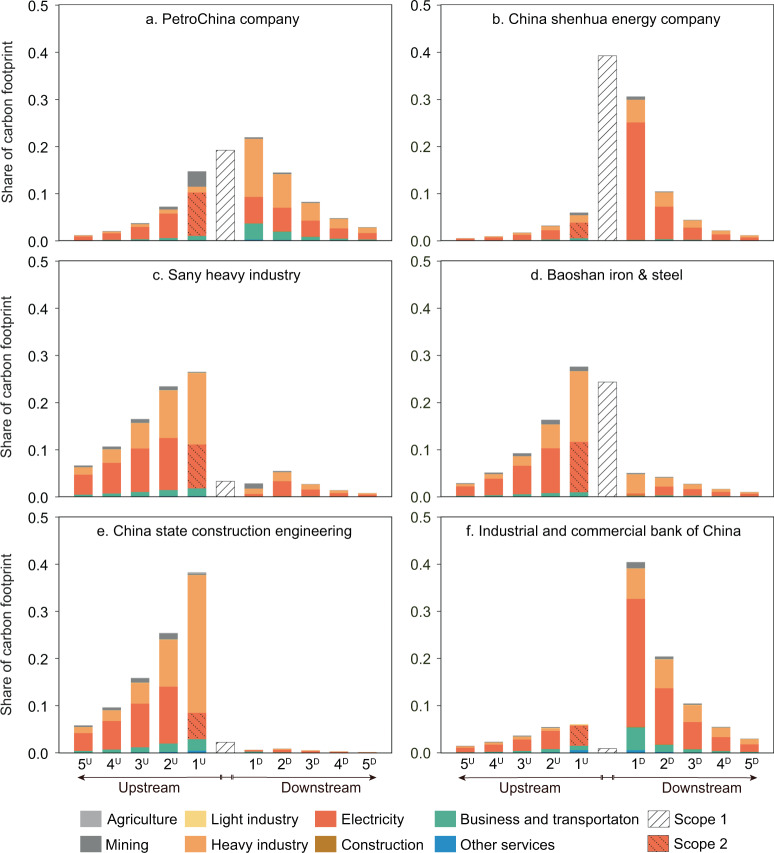
Distribution of carbon footprints along value chains. Panels **a**–**f** show the distribution of carbon footprints of the representative companies in the petroleum, coal, equipment manufacture, steel, construction, and financial sectors, respectively. For every single panel, the bar chart in the middle represents the share of Scope 1 CO_2_ emissions of each firm. The five bars on the left and on the right represent the share of indirect emissions embodied in the upstream and downstream five tiers along the value chain, respectively.

The structure of a company’s carbon footprint is reflected not only by the difference between the volumes of its upstream and downstream value chain carbon footprint but also by the distribution of carbon footprints along different tiers of the value chain. The proposed method allows us to trace carbon footprints in each tier of the value chain^25^. According to Fig. 2, we identified six key sectors with large volumes of upstream or downstream carbon footprints, that is, petroleum, coal, equipment manufacture, steel, construction, and financial sector. Then, we present the distribution of the carbon footprints of the representative companies in these six sectors. The volume of carbon emissions tends to decrease with the increase in the number of tiers. The upstream suppliers in all five tiers account for an average of 90.7% of upstream value chain carbon footprints, and the share is 92.5% for downstream value chain carbon footprints. According to the Pareto rule, which is widely used in the Life Cycle Analysis^26^, Fig. 3 only presents the distribution of carbon footprints with five tiers of value chains.

PetroChina is China’s largest oil and gas producer and emits a large amount of CO_2_ emissions during the production process. Figure 3 shows that Scope 1 emissions account for 19.1% of the gross carbon footprint. PetroChina relies on upstream mining and electricity companies, and its products are necessary to support the production activities of downstream users, such as manufacturing companies. Therefore, the volume of emissions embodied in the upstream and downstream tiers of PetroChina’s value chain is also large, and its carbon footprint peaks at the first downstream tier of its value chain. Similarly, the first-tier downstream electricity companies of China Shenhua Energy, which is China’s largest coal producer, correspond to a large share of this company’s carbon footprint, as its coal resources are used mainly by downstream electricity producers. The business of China Shenhua Energy also incorporates electricity generation, which involves the direct combustion of fossil fuels. Therefore, the Scope 1 emissions of China Shenhua Energy represent a large portion of its total carbon footprint, reaching as high as 39.2%.

Sany Heavy Industry is a magnate in the heavy construction machinery industry in China. Its direct emissions are limited, yet its production activities rely on intermediate inputs from upstream suppliers. In the first upstream tier of its value chain, the heavy industry and electricity generation sector correspond to the highest share of carbon footprints. It should be noted that the volume of CO_2_ emissions embodied in its sub-tier suppliers is also adequate. For instance, the second upstream tier of its upstream value chain corresponds to 23.4% of its gross carbon footprint. This highlights that cumulative climate actions along the value chain could not only be limited to first-tier suppliers but also extend to sub-suppliers. Iron production is carbon intensive and relies on upstream raw materials. Hence, the share of Scope 1 emissions is high, as are the emissions embodied in upstream suppliers. Baoshan Iron & Steel, the major steel supplier in the Chinese market, can reduce its carbon footprint by improving the production process and energy efficiency. Analogously, the construction company relies on the construction materials supplied by upstream value chain partners; therefore, the upstream carbon footprints account for a large share of the carbon footprint of China State Construction Engineering (95.1%). In contrast, as the main investment body of production companies, the carbon footprint of the Industrial and Commercial Bank of China (ICBC), a large state-owned bank managed by the central government, is concentrated mainly in the downstream value chain, especially in the electricity generation sector.

### Financed emissions of leading asset managers’ equity portfolio investment

**Fig. 4:**
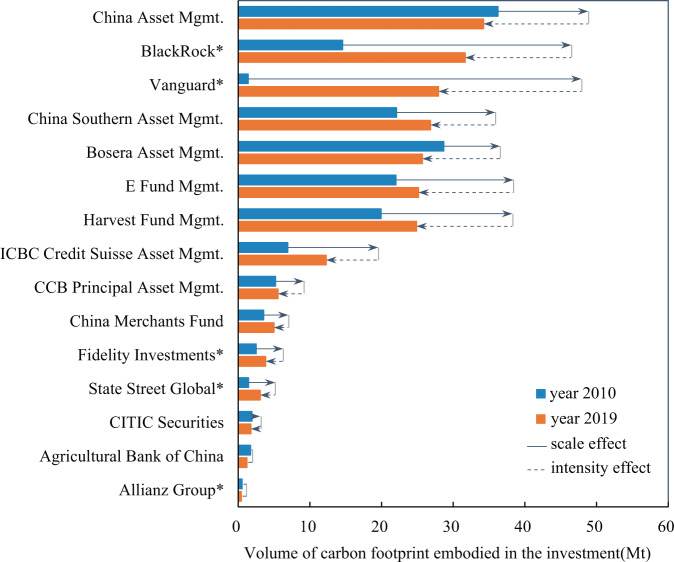
Carbon footprint of leading asset managers’ equity portfolio investment in China’s stock market. The bar indicates the volume of the carbon footprint embodied in the investment of the asset manager, while different colors indicate different years. The solid line arrow and dotted line arrow represent the change in the volume of the carbon footprint due to the scale effect and intensity effect, respectively. The longer the line is, the greater the effect. Companies labeled with a superscript “*” are foreign asset managers.

Information on the value chain carbon footprints of listed companies not only provides a basis for their own emission reduction but also informs the investment strategies of financial institutions. Financial institutions shall account for all investees’ Scope 1, 2 and 3 emissions from 2026 onward^21^. The database on the value chain carbon footprints of Chinese listed companies could help financial institutions gain early insight into the emissions financed by their equity portfolio investments. Figure 4 presents the financed emissions of the equity portfolio investments of the top ten leading domestic and top five leading foreign asset managers in 2019. The ranking of these asset managers is obtained from Thinking Ahead Institute^27^.

Figure 4 shows that there are significant differences in the volume of financed emissions of different asset managers. In 2019, China Asset Mgmt. held the largest amount of financed emissions (34.2 million tonnes) among the fifteen asset managers, while that of Allianz Group was only 0.4 million tonnes. The primary reason for this difference is that the former has a greater investment scale than the latter in the Chinese stock market (Supplementary Information 3.3). The volume of financed emissions is also related to the investment structure. For instance, the investment scale of Bosera Asset Mgmt. is only 1.2 times larger than that of ICBC Credit Suisse Asset Mgmt. However, Bosera Asset Mgmt. tends to invest more in fossil fuel extraction and heavy industries such as metal smelting and rolling, while the financial sector accounts for a large portion of the investment of ICBC Credit Suisse Asset Mgmt. Thus, the former has twice as many financed emissions as the latter. In addition, the gap in the financed emission intensity of these two asset managers reached 46.2 t/million yuan.

Over the past decade, there has been an increase in financed emissions for most asset managers, and the emissions embodied in foreign asset managers’ investment in China’s stock market have increased even more rapidly because China’s stock market has been opening up to international investment over the past decade. The financed emissions and investment scale of Vanguard, in particular, have increased twenty-fold over the past decade. Although the scale effect positively contributes to the increase in financed emissions of asset managers, the intensity effect contributes to a decline in financed emissions. The financed emission intensity of asset managers has shown a declining trend over the past decade, and foreign asset managers correspond to a much sharper decrease (Supplementary Information 1.5). For instance, the financed emission intensity of Vanguard decreased from 386.6 t/million yuan in 2010 to 225.8 t/million yuan in 2019. We review the green investment principles that asset managers follow (Supplementary Information 3.4) and find that foreign asset managers are building portfolios aligned with climate change concerns. For instance, Fidelity Investment, which has actively signed initiatives or joined groups such as Climate Action 100+ (Supplementary Information 3.4), has withdrawn nearly half of its investment from carbon-intensive sectors, especially chemicals, and increased its investment in sectors with lower carbon emissions, such as the manufacturing sectors, in the past decade (Supplementary Information 3.3). The trend of shifting financial flows to low-carbon companies is relatively small for domestic asset managers. China Asset Mgmt., for example, has reduced its investments in the coal mining and chemical industries but increased its investments in the electricity generation and transportation industries, which are also carbon-intensive industries.

## Discussion

The carbon footprints of listed companies have attracted social attention over the past few years. For instance, Caijing magazine published the top 100 list of Chinese listed companies by volume of direct emissions in 2021^28^. As the volume of indirect emissions is significantly greater than that of direct emissions, the present study shifts attention from the direct to indirect emissions associated with listed companies. The United States Securities and Exchange Commission has stipulated that listed companies have to report their direct emissions, as well as indirect emissions, as appropriate by 2026. Disclosing the value chain carbon footprints of listed companies could be a major trend in the near future. The present study could help Chinese listed companies adapt to this trend by introducing a supplementary top-down measurement framework and building a database for the carbon emissions embodied in the value chains of listed companies over the period 2010–2019. Future studies could further apply the proposed measurement framework to other public companies in other countries.

The homogeneity assumption of the EIO-LCA method used in this paper determines that the method may be less specific for an individual company^29^. Yet, estimation of the value chain carbon footprints based on industry-average data may actually be more accurate than process LCA^2^, which is complex^30^ and unsystematic^31^. Future studies could further extend the present study by adopting input‒output tables that distinguish different production actions^32, 33^. For instance, the Analytical AMNE database provides an input‒output table that captures firm heterogeneity. Zhang et al.^34^ adopt the Analytical AMNE database to calculate the carbon footprints of domestic and foreign-owned companies. Future studies could also apply the proposed method to the Analytical AMNE database to trace the carbon footprints of overseas operations of Chinese listed companies. In addition, future studies could adopt the hybrid LCA method^35^, which combines EIO-LCA and process LCA, to reflect the difference in production technologies among listed companies.

The Paris Agreement set the finance flow goal of shifting finance flows to more climate-friendly and climate-resilient activities. The decarbonization of investment activities is another trend in the field of climate change mitigation^36^. To continuously attract financial support from the stock market, listed companies need to set ambitious climate targets and adopt active climate actions regarding their value chain carbon footprints. To help listed companies adopt more targeted climate actions, the present study not only provides the volume of the value chain carbon footprints of listed companies but also clarifies the distribution of carbon footprints along the value chain. Companies with a greater volume of upstream/downstream value chain carbon footprints need to cooperate with their upstream suppliers/downstream users to reduce their carbon footprints. In addition, the results of the present study could help investors understand their climate risk across their value chains.

There may be several factors affecting the structural carbon footprints of listed companies, such as their position in the value chain and industry attributes. We measure the position of an agent in the domestic value chain in China based on the degree of upstreamness and analyze the impact of industry attributes and position in the value chain on the structure of carbon footprints. Supplementary Information 1.3 shows that the degree of upstreamness is negatively related to the share of upstream value chain carbon footprints and is positively related to the share of downstream value chain carbon footprints. An enterprise located upstream (downstream) of domestic value chains tends to have a greater share of downstream (upstream) value chain carbon footprints. For instance, mining companies tend to have a greater volume of downstream value chain carbon footprints, and construction companies tend to have a greater volume of upstream value chain carbon footprints. Other factors, such as location, ownership characteristics, and market capitalization, may also influence the structural carbon footprints of listed companies. Future studies could further explore these impacts.

Several other potential extensions are worth pursuing in future studies. First, this study evaluates only the financed emissions of leading asset managers’ equity portfolio investments. However, there are also other types of investors. For instance, individual investors account for an enormous share of China’s stock market. Future studies could analyze the financial emissions of other types of investors. Second, listed companies need to disclose not only their carbon footprints but also their climate risk. The results of the present study could provide a foundation on which future studies could evaluate the climate risk of listed companies and their investors. For instance, the greater the carbon emissions embodied in the investee’s production activities are, the higher the investor’s exposure to climate policy risks^37^. Third, a company’s control power over its own value chain is also influenced by other factors, such as the length and complexity of the production networks^38^. Future studies are expected to take these factors into account to provide more reasonable suggestions on how to mitigate value chain carbon footprints.

## Methods

There are bottom-up methods^39^ and top-down methods to quantify firms’ carbon footprints. The bottom-up method is complex^30^, unsystematic^31^ and unusable if the number of studied firms is large. In contrast, the top-down method, which is based on the EIO-LCA model^40^, has the advantages of a complete system boundary and comparable accounting results among different entities and is more convenient and practical for large-scale use. The EIO-LCA is also accepted by the IPCC^41, 42^. This paper proposes an EIO-LCA method to calculate carbon emissions at the company level.

According to the Leontief demand-driven IO model, the output vector $X$ is determined by the final demand vector $Y$.

1 $$X={(I-A)}^{-1}Y={BY}$$

where $A$ is the intermediate input matrix and $B$ is the Leontief inverse matrix. We define the direct carbon intensity matrix as $F$, which is a diagonal matrix, and the elements are the carbon emissions intensity of each sector. The carbon emissions generated in the production process are denoted as ${FBY}$. $V$ is a diagonalized matrix of the value-added ratio. Since the column sum of matrix$\,{VB}$ is equal to 1, ${VB}$ can be observed as an allocation matrix. By multiplying ${VB}$ and ${FBY}$, we can trace both demand- and supply-driven carbon emissions^11^.

Based on the hypothetical extraction approach^34^, the emissions related to the production activity of sector $i$ in region ${r}$ are

2 $${E}_{{ri}}={VBFBY}-{V}_{{ri}}^{*}{B}_{{ri}}^{*}{F}_{{ri}}^{*}{B}_{{ri}}^{*}{Y}_{{ri}}^{*}$$

where ${V}_{{ri}}^{*}$ is the value-added ratio matrix that has removed the elements that are related to sector $i$ in region ${r}$. ${B}_{{ri}}^{*}={(I-{A}_{{ri}}^{*})}^{-1}$, and ${A}_{{ri}}^{*}$ is the intermediate input matrix that has removed the elements that are related to sector $i$ in region ${r}$. ${Y}_{{ri}}^{*}$ is the final demand matrix that has removed the elements that are related to sector $i$ in region ${r}$. ${F}_{{ri}}^{*}=F-{F}_{{ri}}$, where ${F}_{{ri}}$ is the carbon intensity matrix that is made up of only the carbon intensity coefficient of sector $i$ in region ${r}$. The carbon footprints can be divided into different parts.

3 $${E}_{ri}=	\underbrace{{VBF}_{ri}{BY}}_{{3.1}\,{direct}\,{emissions}({Scope}\,{1})} \\ 	+ \,\underbrace{ {\,} \underbrace{{VBF}_{ri}^{\ast }({BY}-{B}_{ri}^{\ast }{Y}_{ri}^{\ast })}_{{3.2.1}\,{upstream}\,{emissions}}+\underbrace{(VB-{V}_{ri}^{\ast }{B}_{ri}^{\ast }){F}_{ri}^{\ast }BY}_{{3.2.2}\,{downstream}\,{emissions}}-\underbrace{(VB-{V}_{ri}^{\ast }{B}_{ri}^{\ast }){F}_{ri}^{\ast }(BY-{B}_{ri}^{\ast }{Y}_{ri}^{\ast })}_{{3.2.3}\,duplication\,part}}_{{3.2}\,{indirect}\,{emissions}({Scope}\,{2}+{3})}$$

The first part (3.1) represents the direct emissions of sector $i$ in region ${r}$. The second part (3.2) represents the indirect emissions or value chain carbon footprint of sector $i$ in region ${r}$. Subparts (3.2.1) and (3.2.2) represent the indirect upstream and downstream production carbon footprints of sector $i$ in region ${r}$, respectively. It should be noted that the products of sector$\,i$ may be first processed by sector $j$ and then returned to sector $r$ for further processing. In other words, sector $j$ could be observed as being located either upstream or downstream relative to sector ${r}$. The carbon emissions of sector $j$ are counted by both terms (3.1) and (3.2); therefore, equation (3.2) needs to subtract the third term (3.2.3), which represents the duplication of the upstream and downstream carbon footprints. The gross volume of upstream emissions is ${VB}{F}_{{ri}}^{*}({BY}-{B}_{{ri}}^{*}{Y}_{{ri}}^{*})$, which is consistent with the literature^9, 34^. We further define an emission intensity matrix ${F}_{e}$, which is made up of the carbon emission intensity of the electricity and heat generation sector. ${A}_{{ri}}$ is a matrix made up of the intermediate input ratio of sector $i$ in region ${r}$. Then, the term (3.2.1) can be divided into${F}_{e}{A}_{{ri}}{BY}$ (Supplementary Information 2.4) and ${VB}{F}_{{ri}}^{*}({BY}-{B}_{{ri}}^{*}{Y}_{{ri}}^{*})-{F}_{e}{A}_{{ri}}{BY}$. The former represents the emissions associated with the purchased electricity and heat of sector $i$ in region ${r}$, and the latter is the upstream Scope 3 carbon footprint.

By decomposing the term (3.2.1), we can trace the emissions embodied in upstream production stages that directly or indirectly supply intermediate inputs to support the production of sector ${i}$.

4 $${VB}{F}_{{ri}}^{*}\left({BY}-{B}_{{ri}}^{*}{Y}_{{ri}}^{*}\right)={VB}{F}_{{ri}}^{*}(I+{A}_{{ri}}^{*}+{A}_{{ri}}^{*2}+\cdots ){X}_{{ri}}$$

The derivation of Eq. (4) is presented in Supplementary Information 2.1. For instance, ${VB}{F}_{{ri}}^{*}I{X}_{{ri}}$ represents the emissions of the first-tier suppliers of sector ${i}$, and ${VB}{F}_{{ri}}^{*}{A}_{{ri}}^{*}{X}_{{ri}}$ represents the emissions of the second-tier suppliers of sector ${i}$. We decompose the term (3.2.2) in Eq. (3) **(**please refer to Supplementary Information 2.2) to trace the emissions embodied in downstream production stages that directly or indirectly use the intermediate inputs supplied by this sector.

5 $$({VB}-{{V}_{{ri}}^{*}{B}_{{ri}}^{*}F})_{{ri}}^{*}{BY}=({V}_{{ri}}+{VB}{A}_{{ri}})(I+{A}_{{ri}}^{*}+{A}_{{ri}}^{*2}+\cdots ){F}_{{ri}}^{*}X$$

Here, the gross outputs of sector $i$ in region ${r}$ are extracted (Supplementary Information 1.1 and 1.2). We can also extract part of the outputs from the IO model^9^. We collect listed companies’ revenue at the regional and sectoral levels from their annual reports and then assign revenue items from the annual reports to the sectors and regions of the IO table. Then, by partially extracting the corresponding outputs that are the same as the revenue of a company from the IO model, we can obtain the carbon footprint of the company. Similarly, we can obtain the emissions financed by an investor based on the share of the listed company’s market capitalization owned by this investor.

The National Bureau of Statistics of China publishes the provincial IO table every 5 years. Here, we adopt the multiregional input‒output table for 2012 and 2017. We assume that there is no significant change in economic structure and production technology in the 2 years before and after 2017, and the same applies for 2012. Therefore, we apply the multiregional input‒output table for 2012 and 2017^43^ (Supplementary Information 3.1 and 3.2) to calculate the carbon footprints of listed companies over the periods 2010–2014 and 2015–2019, respectively. The annual carbon emissions data are from the Carbon Emission Accounts and Datasets for emerging economies(CEADs)^16, 44^.

### Reporting summary

Further information on research design is available in the Nature Portfolio Reporting Summary linked to this article.

## Supplementary information

Supplementary information

Reporting Summary

## Data Availability

The data that support the findings of this study are provided in the Supplementary Information. Other data have been deposited in Figshare (10.6084/m9.figshare.21936938.v2). The multiregional input-output (MRIO) table used in this study is available at 10.1021/acs.est.8b03424. The annual carbon emissions data used in this study is available at the Carbon Emission Accounts and Datasets for emerging economies (CEADs) (https://www.ceads.net.cn/data/province/). The list of Chinese listed companies is available at China Stock Market & Accounting Research Database (CSMAR) (http://cndata1.csmar.com). The operating information of Chinese listed companies is obtained from their annual reports.

## Code availability

The code to calculate value chain carbon footprints at the sectoral level can be accessed at Figshare (10.6084/m9.figshare.21936938.v2).
