# Supplementary material for: Value chain carbon footprints of Chinese listed companies
Source: Nat Commun. 2023 May 16;14:2794. doi: 10.1038/s41467-023-38479-5 (PMC10188601; doi:10.1038/s41467-023-38479-5)
Supplement: Supplementary file 1 — Supplementary information [file 41467_2023_38479_MOESM1_ESM.pdf]

Supplementary Information for

Value chain carbon footprints of Chinese listed companies

Zengkai Zhang[1], Jiaoyan Li[2], Dabo Guan[3,4,*]

[1]State Key Laboratory of Marine Environmental Science, College of the Environment and Ecology, Xiamen University, Xiamen, Fujian, 361102, China.
[2]College of Management and Economics, Tianjin University, Tianjin 300072, China.
[3]Department of Earth System Science, Tsinghua University, Beijing, 100084, China.
[4]The Bartlett School of Construction and Project Management, University College London, London, WC1E 7HB, UK.

Contents

1. Supplementary information on the calculation results S2
1.1. Structure of carbon footprints at the provincial and sectoral levels S2
1.2. The distribution of carbon footprints along the value chain S3
1.3. Value chain position and value chain carbon footprint S6
1.4. Direct and indirect carbon footprints of Chinese listed companies S7
1.5. Carbon intensity of leading asset managers' equity portfolio investment S8
2. Supplementary information on the calculation methods S9
2.1. The derivation of equations (4) S9
2.2. The derivation of equations (5) S9
2.3. Method to calculate emissions related to Chinese listed companies S9
2.4. An example to explain how to account for the Scope 2 carbon footprint S10
3. Supplementary information on the data S11
3.1. 42 sectors of the input-output tables S11
3.2. 31 provinces of the input-output tables S12
3.3. Leading asset managers' equity portfolio investment in China's stock market S13
3.4. The green investment principles that asset managers follow S15

1. Supplementary information on the calculation results

1.1. Structure of carbon footprints at the provincial and sectoral levels

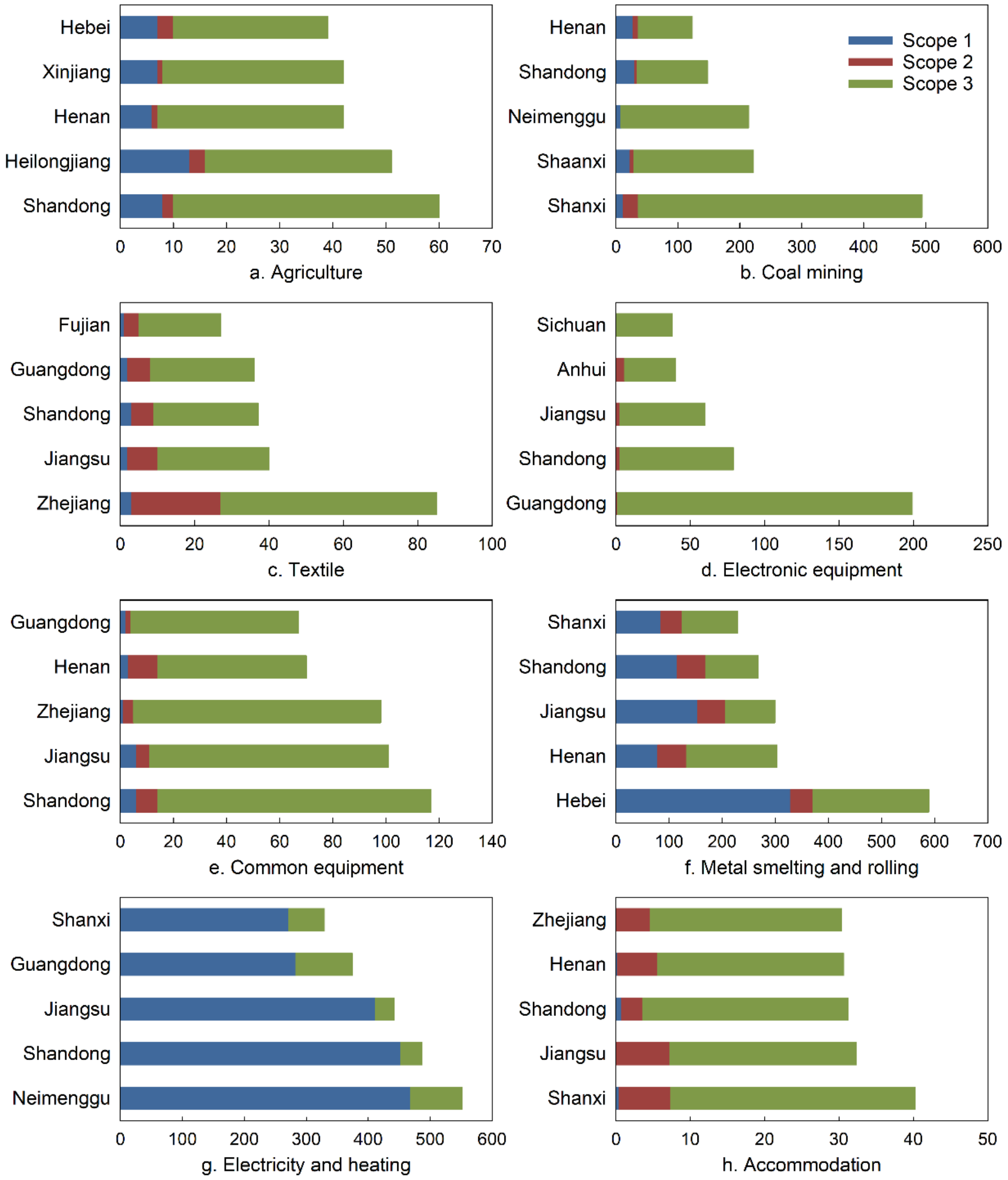

Fig. S1| Scope 1, 2, and 3 carbon footprints at the provincial and sectoral levels in 2017 (million tonnes)

Fig. S1 shows the results on the carbon footprints of eight sectors in the top five provinces by the volume of carbon footprints. There exists significant regional and sectoral heterogeneity in the structure of carbon footprints. The Scope 1 carbon footprint corresponds to a relatively greater share for energy-intensive sectors, such as the electricity and heating sector (Fig S1.g) and metal smelting and rolling sector (Fig. S1.f), which generate CO_2 emissions to support the

production activities of other sectors. For the other six subfigures, their carbon footprint is made up mainly of indirect emissions (Scope 2 and 3 carbon footprints). For example, the business activities in the accommodation sector rely on electricity and heat supply. Therefore, the Scope 2 carbon footprints related to electricity and heat supply account for a greater share. In addition to the electricity and heat supply sector, many other agents, whose emissions are related to Scope 3 carbon footprints, are involved in the upstream and downstream value chains. Figure 1 shows that the volume of the Scope 3 carbon footprint tends to be significantly greater than that of the Scope 2 carbon footprint. For instance, the carbon footprint of the electronic equipment sector is composed mainly of the CO_2 emissions embodied in the upstream and downstream value chains. The huge volume of a sector's Scope 3 carbon footprint highlights that firms in this sector have a greater influence on reducing other firms' carbon emissions and have greater collaborative opportunities in terms of climate actions with their value chain partners. Therefore, it is essential for these firms to engage in value chain climate change mitigation activities to promote collaborative climate actions along the value chain.

Developed coastal provinces, such as Guangdong and Zhejiang, and the inland fossil-fuel producing provinces, such as Shanxi and Neimenggu, tend to have a large carbon footprint. These provinces all hold critical positions in China’s domestic production networks. Their climate actions would have a powerful demonstration effect on other regions. China’s climate policies towards greener value chains should be more targeted towards these provinces. Although the present study presents only the results of the top five provinces, the structures of their carbon footprints are almost the same. The structure of a sector’s carbon footprint is determined mainly by the position of this sector in the production networks. For instance, those sectors located upstream of the production network tend to correspond to greater downstream Scope 3 emissions and vice versa. An increasing number of enterprises, such as Microsoft[1], are adopting climate measures to reduce not only their direct emissions but also their value chain emissions. The information on the sectoral carbon footprint could also help provide a more complete picture of the structure of those CO_2 emissions that are directly and indirectly related to enterprises' production activities. In addition, the carbon disclosure behavior of most firms is voluntary[2], and these firms may adopt different approaches and boundaries[3]. The results for firms within the same sector may not be comparable. The sector information could also be used as a benchmark by firms to determine where they should focus their emission mitigation efforts.

1.2. The distribution of carbon footprints along the value chain

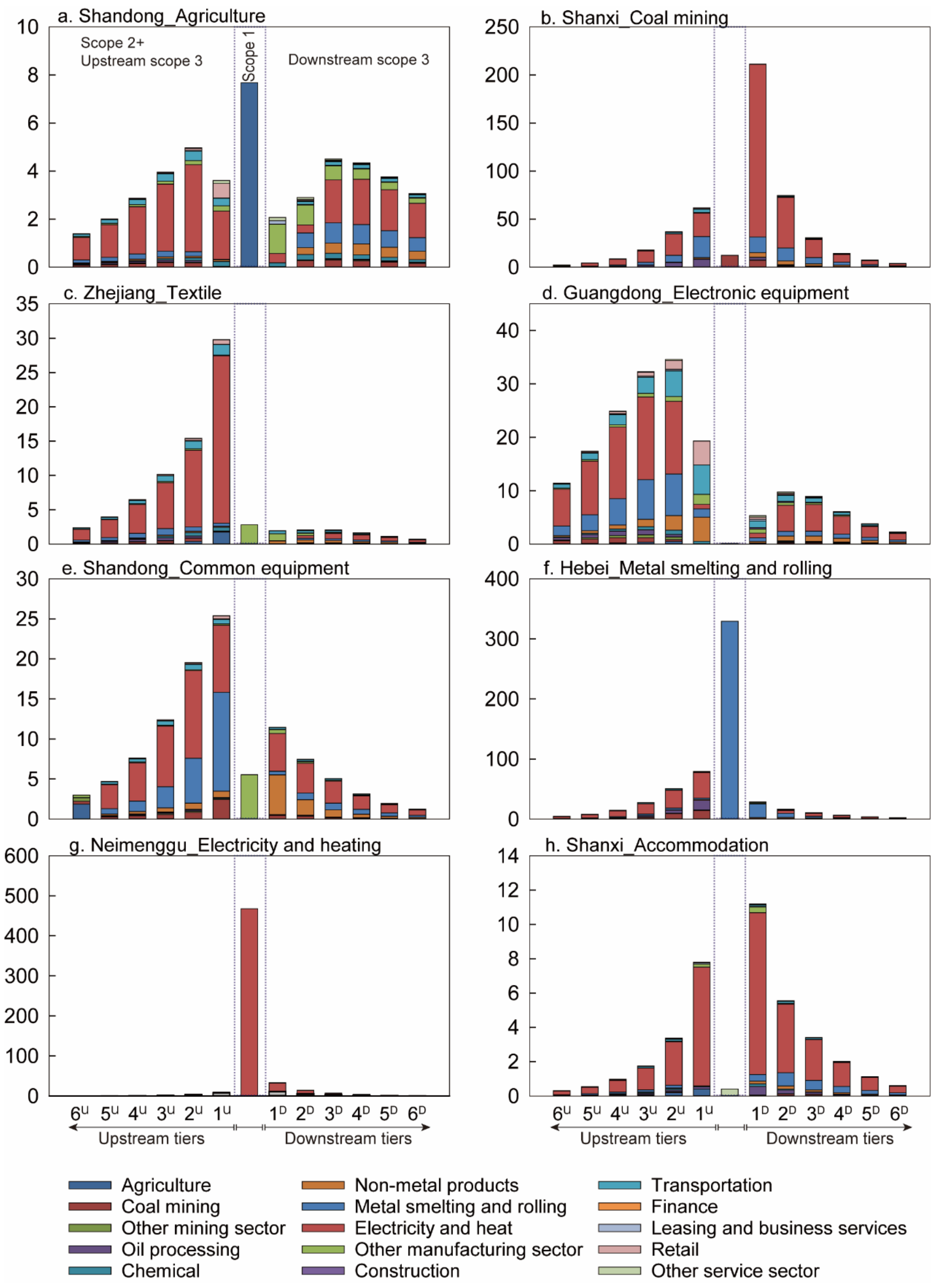

Fig. S2| The distribution of carbon footprints along value chains. Panels **a-h** show the distribution of carbon footprints of agriculture, coal mining, textile, electronic equipment, common equipment, metal smelting and rolling, electricity and heating, accommodation in provinces that hold the largest volume of carbon footprints in corresponding sectors, respectively. Each panel is divided into three areas. The bar in the middle area represents the scope 1 emissions. The bars in the left area represent the scope 2 emissions and upstream scope 3 emissions. The bars in the right area represent the downstream scope 3 emissions. The tiers of upstream suppliers and downstream users are labeled by the superscript U and

D , respectively.

The indirect carbon footprints are made up of emissions embodied in the economic activities of upstream suppliers and downstream users. Fig. S2 shows that, for the manufacturing sectors, the volume of carbon emissions embodied in the upstream suppliers account for a greater share in the gross carbon footprints. For instance, the emissions embodied in the upstream supply chains of the textile sector in Zhejiang province account for 84.5% of its gross carbon footprint. The production activities of the downstream users of coal mining sectors would result in a huge volume of carbon emissions, which highlights that the carbon mitigation strategies on the coal mining sector should focus on the carbon emissions enabled by the extraction activities of coal mining enterprises[4]. The agriculture and accommodation sectors have a great volume of upstream and downstream indirect emissions, and enterprises in these two sectors need to cooperate with both upstream and downstream suppliers to reduce their carbon footprints.

The indirect carbon footprints are related to different economic activities[5], such as business travel, commodity transportation, and investment. Fig. S2 aggregates these economic activities into fifteen sectors and shows the volume and structure of sectoral carbon footprints in different tiers of value chains. The tier of the value chain corresponding to the largest share of carbon footprint highlights the potential opportunities for firms to reduce their environmental impacts by rearranging the value chain that they are involved in. The volume of carbon emissions embodied in different tiers of the value chain is not necessarily peak at the first tier from both upstream and downstream perspectives. For instance, the second upstream tier of the electronic equipment sector's value chain in the Guangdong province corresponds to the greatest volume of embodied carbon emissions. This highlights that climate policies should encourage enterprises to cooperate with not only the first-tier value chain partners but also the other agents that are involved in the value chain. An effective climate policy should cover carbon emissions embodied in the value chain across multiple tiers. The structure of the carbon footprints at each tier could help the related enterprises to find the major value chain partners for reducing carbon footprint. Fig. S2 shows that the emissions embodied in the electricity generation sector correspond to the largest share in the different tiers of carbon footprints. The volume of emissions embodied in the indirect electricity use may be greater than the volume of emissions embodied in their purchased electricity. Enterprises should not only use a greater share of green electricity but also encourage other value chain partners to use green electricity. In addition, the metal smelting and rolling sector also accounts for a greater share in the carbon footprint of manufacturing sectors and the climate actions of the metal smelting and rolling sector would contribute to the development of green value chains of the manufacturing enterprises.

1.3 Value chain position and value chain carbon footprint

This study measures the position of an agent in the domestic value chain in China based on the degree of upstreamness. The calculation is based on the method ($\frac{VBBy}{VBy}$) which is widely adopted by the literature [6–8]. Here, we present the relation between the position of the value chain and the structure of value chain carbon footprints.

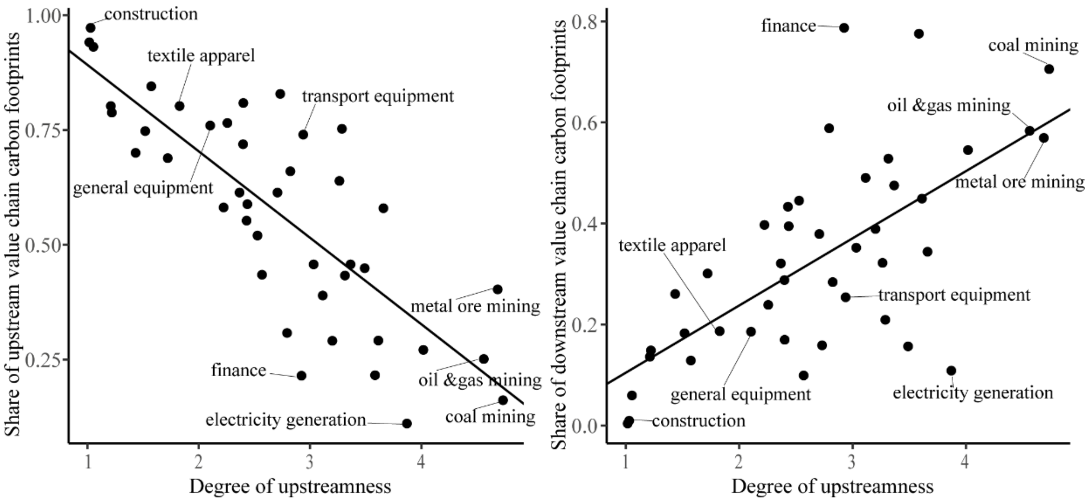

Fig. S3| The relation between the degree of upstreamness and the structure of value chain carbon footprint. Notes, each point in Figure S3 represents a sector.

Figure S3 shows that the degree of upstreamness is negatively related to the share of upstream value chain carbon footprints and is positively related to the degree of the share of downstream value chain carbon footprints. This means that the structure of an agent's carbon footprint is closely related to the location of this agent in the value chain. An enterprise located in the upstream (downstream) of domestic value chains tends to have a greater share of downstream (upstream) value chain carbon footprints. For instance, mining companies tend to have a greater volume of downstream value chain carbon footprints, and construction companies tend to have a greater volume of upstream value chain carbon footprints. The production activities of transport equipment companies rely on materials from upstream suppliers. The emissions embodied in supply chains should be the focus of climate policies on transport equipment companies to reduce their carbon footprints. The situation is just the opposite for the coal mining sector. The downstream users buy coal resources from coal mining enterprises as fuels or raw materials. The production activities of the downstream users would result in a huge volume of carbon emissions.

1.4 Direct and indirect carbon footprints of Chinese listed companies

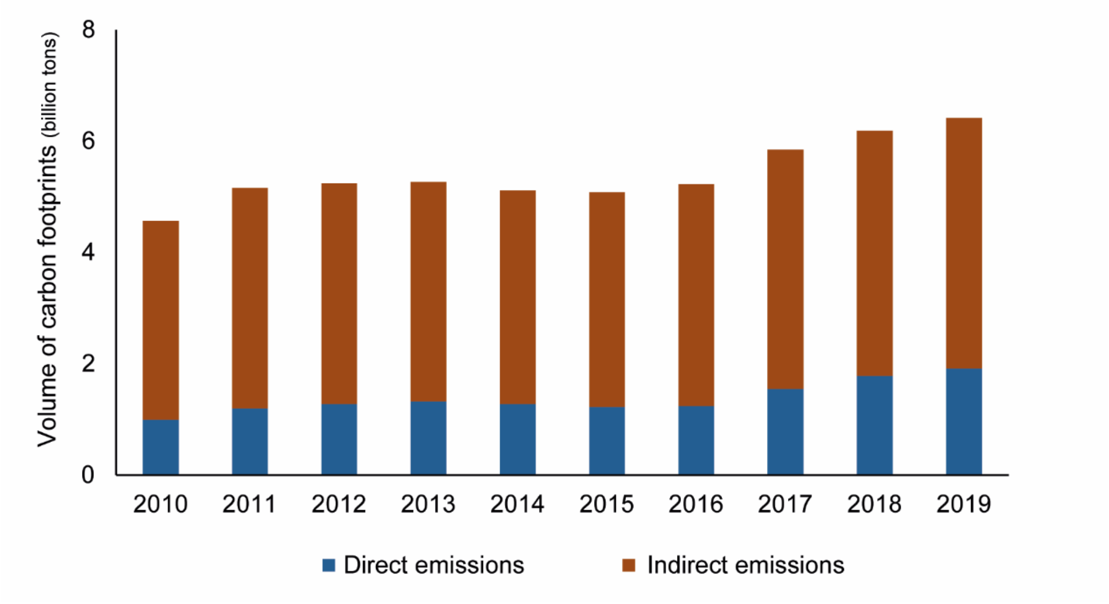

Fig. S4| Carbon emissions embodied in the whole value chains of listed companies.

The volume of direct emissions emitted by Chinese listed companies has been increasing over the past decade, accounting for 18.3% of national emissions in 2019. The indirect emissions emitted to support the production activities of these companies are well over two to three times larger than the direct emissions over the 2010 to 2019 period. It is crucial to draw attention to reducing indirect emissions of listed companies. Their great control power over the entire value chain could help speed up the climate mitigation activities of other unlisted companies and make a huge difference to the progress of realizing Chinese carbon reduction targets.

1.5 Carbon intensity of leading asset managers' equity portfolio investment

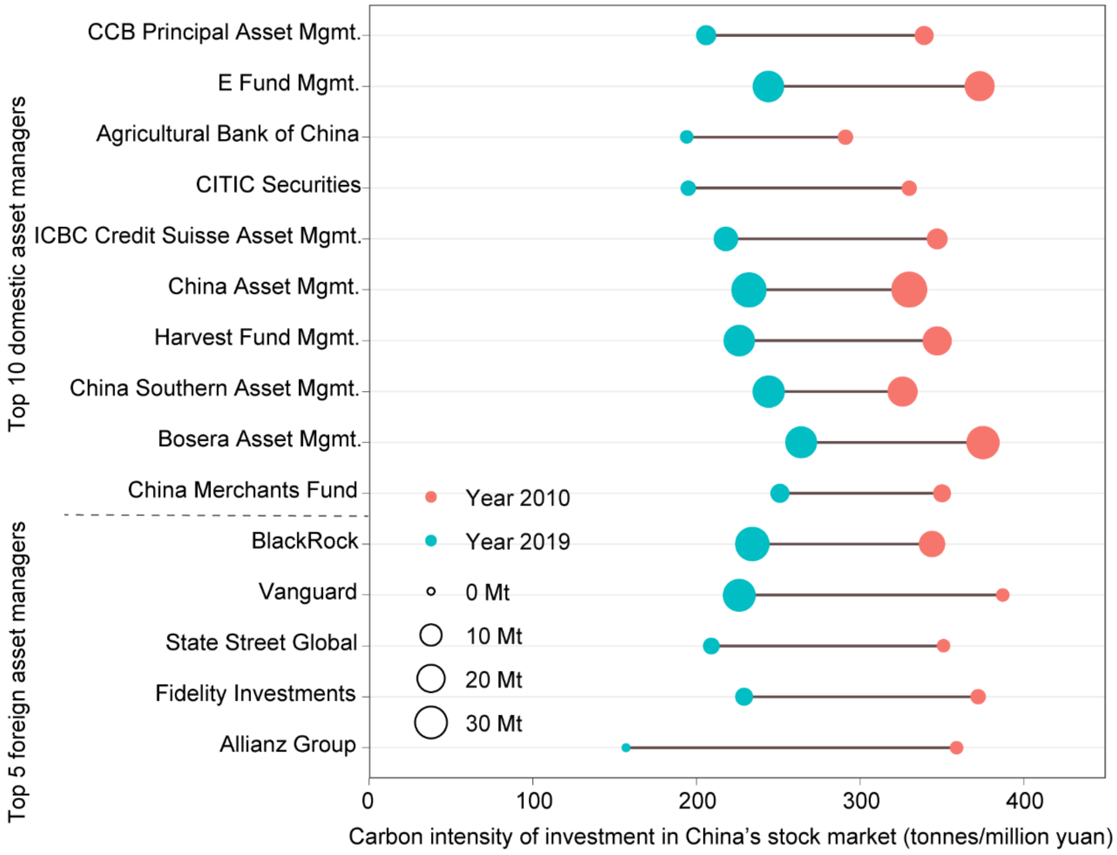

Fig. S5| Carbon intensity of leading asset managers' equity portfolios investment in China's stock market. The size of the bubble indicates the carbon footprint embodied in the investment of the asset manager, while different colors indicate different years. The solid line connecting the two bubbles represents the change in carbon intensity, and the length represents the degree of such change. The longer the line is, the greater the change.

The carbon footprint intensity of different asset managers is determined mainly by their investment structure. For example, service accounts for a large portion of the investment of the Agricultural Bank of China, while Bosera Asset Mgmt. tends to invest more money in carbon-intensive industries, such as the electricity generation industry, which leads to a gap of 70.7 t/million yuan. The financed emission intensity of asset managers has shown a declining trend over the past decade, and foreign asset managers correspond to a much sharper decrease. For instance, the financed emissions intensity of Allianz Group decreased from 359.3 t/million yuan in 2010 to 157.3 t/million yuan in 2019. Moreover, Allianz Group has withdrawn a large amount of its investment from carbon-intensive industries such as coal mining, metal smelting and rolling industries and increased its investment in industries with lower carbon emissions such as the leasing and business services industries in the past decade. The trend of shifting financial flows to low-carbon companies is relatively small for domestic asset managers. China Asset Mgmt., for example, has reduced its investments in coal mining and chemical industries but

increased its investments in electricity generation and transportation industries, which are also carbon-intensive industries. It is necessary to implement stricter measures to encourage asset managers to shift financial flows to low-carbon fields, such as increasing the risk weight of carbon intensive assets and discounting interest on green credit products.

2. Supplementary information on the calculation methods

2.1. The derivation of equations (4)

$$
\begin{aligned}
& VBF_{ri}^{*}\left(BY-B_{ri}^{*}Y_{ri}^{*}\right)=VBF_{ri}^{*}\left(\left(I+A+A^{2}+\cdots\right)Y-B_{ri}^{*}Y_{ri}^{*}\right)\\
&=VBF_{ri}^{*}\left(\left(I+A+A^{2}+\cdots\right)\left(Y_{ri}+Y_{ri}^{*}\right)-B_{ri}^{*}Y_{ri}^{*}\right)\\
&=VBF_{ri}^{*}\left(\left(I+\left(A_{ri}^{*}+A_{ri}\right)+\left(A_{ri}^{*}+A_{ri}\right)^{2}+\cdots\right)\left(Y_{ri}+Y_{ri}^{*}\right)-B_{ri}^{*}Y_{ri}^{*}\right)\\
&=VBF_{ri}^{*}\left(\left(B_{ri}^{*}+B_{ri}^{*}A_{ri}B_{ri}^{*}+B_{ri}^{*}A_{ri}B_{ri}^{*}A_{ri}B_{ri}^{*}+\cdots\right)\left(Y_{ri}+Y_{ri}^{*}\right)-B_{ri}^{*}Y_{ri}^{*}\right)\\
&=VBF_{ri}^{*}\left(\left(B_{ri}^{*}+B_{ri}^{*}A_{ri}B_{ri}^{*}+B_{ri}^{*}A_{ri}B_{ri}^{*}A_{ri}B_{ri}^{*}+\cdots\right)Y_{ri}+\left(B_{ri}^{*}A_{ri}B_{ri}^{*}+B_{ri}^{*}A_{ri}B_{ri}^{*}A_{ri}B_{ri}^{*}+\cdots\right)Y_{ri}^{*}\right)\\
&=VBF_{ri}^{*}B_{ri}^{*}Y_{ri}+VBF_{ri}^{*}B_{ri}^{*}A_{ri}X\\
&=VBF_{ri}^{*}B_{ri}^{*}\left(Y_{ri}+A_{ri}X\right)\\
&=VBF_{ri}^{*}B_{ri}^{*}X_{ri}\\
&=VBF_{ri}^{*}\left(I+A_{ri}^{*}+A_{ri}^{*2}+\cdots\right)X_{ri}
\end{aligned}
$$

2.2. The derivation of equations (5)

$$
\begin{aligned}
& \left(VB-V_{ri}^{*}B_{ri}^{*}\right)F_{ri}^{*}BY=\left(V\left(I+A+A^{2}+\cdots\right)-V_{ri}^{*}B_{ri}^{*}\right)F_{ri}^{*}BY\\
&=\left(\left(V_{ri}+V_{ri}^{*}\right)\left(I+\left(A_{ri}^{*}+A_{ri}\right)+\left(A_{ri}^{*}+A_{ri}\right)^{2}+\cdots\right)-V_{ri}^{*}B_{ri}^{*}\right)F_{ri}^{*}BY\\
&=\left(\left(V_{ri}+V_{ri}^{*}\right)\left(B_{ri}^{*}+B_{ri}^{*}A_{ri}B_{ri}^{*}+B_{ri}^{*}A_{ri}B_{ri}^{*}A_{ri}B_{ri}^{*}+\cdots\right)-V_{ri}^{*}B_{ri}^{*}\right)F_{ri}^{*}BY\\
&=\left(V_{ri}\left(B_{ri}^{*}+B_{ri}^{*}A_{ri}B_{ri}^{*}+B_{ri}^{*}A_{ri}B_{ri}^{*}A_{ri}B_{ri}^{*}+\cdots\right)+V_{ri}^{*}\left(B_{ri}^{*}A_{ri}B_{ri}^{*}+B_{ri}^{*}A_{ri}B_{ri}^{*}A_{ri}B_{ri}^{*}+\cdots\right)\right)F_{ri}^{*}BY\\
&=\left(V_{ri}B_{ri}^{*}+VBA_{ri}B_{ri}^{*}\right)F_{ri}^{*}BY\\
&=\left(V_{ri}+VBA_{ri}\right)B_{ri}^{*}F_{ri}^{*}BY\\
&=\left(V_{ri}+VBA_{ri}\right)B_{ri}^{*}F_{ri}^{*}X\\
&=\left(V_{ri}+VBA_{ri}\right)\left(I+A_{ri}^{*}+A_{ri}^{*2}+\cdots\right)F_{ri}^{*}X
\end{aligned}
$$

2.3. Method to calculate emissions related to Chinese listed companies

We define $\mathbf{X}_c$ as the output of Chinese listed companies. Then, the direct emissions (scope 1) produced by listed companies can be calculated by $E_{cf\_direct} = FX_c$. Based on the hypothetical extraction method, the emissions that have no relation with listed companies are $E_{cf^*} = V_c^* B_c^* F B_c^* Y_c^*$, in which the letters with superscript "*" and subscript "c" are the matrices that have removed the part of economic activities that are related to listed companies. The

carbon footprint of Chinese listed companies can be then expressed as $E_{cf} = E - E_{cf^*} = VBFBY - V_c^* B_c^* FB_c^* Y_c^*$. Then the indirect emissions (scope 2+3) can be expressed as $E_{cf\_indirect} = E_{cf} - E_{cf\_direct} = (VBFBY - V_c^* B_c^* FB_c^* Y_c^*) - FX_c$. Through tracing the source of the carbon footprint from 42 sectors, we grouped them into eight sectors for clarity. The contribution of direct and indirect emissions to the national emissions in year t can be calculated by $\frac{E_{cf\_direct,t}}{national\ emissions_t}$ and $\frac{E_{cf\_indirect,t}}{national\ emissions_t}$, respectively.

2.4. An example to explain how to account for the Scope 2 carbon footprint

We suppose that there is only one region, which is made up of three sectors. The intermediate input linkages among these three sectors are $A = \begin{bmatrix} a_{11} & a_{12} & a_{13} \\ a_{21} & a_{22} & a_{23} \\ a_{31} & a_{32} & a_{33} \end{bmatrix}$, and the direct carbon intensity matrix is $F = \begin{bmatrix} f_1 & 0 & 0 \\ 0 & f_2 & 0 \\ 0 & 0 & f_3 \end{bmatrix}$. We suppose the sector 3 is the electricity and heat generation sector. $F_1 = \begin{bmatrix} f_1 & 0 & 0 \\ 0 & 0 & 0 \\ 0 & 0 & 0 \end{bmatrix}$ is the direct carbon intensity matrix of sector 1, $F_e = \begin{bmatrix} 0 & 0 & 0 \\ 0 & 0 & 0 \\ 0 & 0 & f_3 \end{bmatrix}$ is carbon intensity matrix of purchased electricity and heat, and $A_1 = \begin{bmatrix} a_{11} & 0 & 0 \\ a_{21} & 0 & 0 \\ a_{31} & 0 & 0 \end{bmatrix}$ is the intermediate input matrix of sector 1. We can obtain that $F_e A_1 = \begin{bmatrix} 0 & 0 & 0 \\ 0 & 0 & 0 \\ f_3 a_{31} & 0 & 0 \end{bmatrix}$, which means the emissions embodied in the purchased electricity and heat to support the production of unit output of sector 1. We can see that F_1 and $F_e A_1$ have different economic meanings, as well as $F_1 BY$ and $F_e A_1 BY$. The former represents the direct emissions of sector 1 (scope 1 carbon footprint), and the latter represents the emissions embodied in purchased electricity and heat of sector 1 (scope 2 carbon footprint). Since the

column sum of matrix VB is equal to 1, the sum of elements of matrix VBF_1BY is equal to that of F_1BY .

3. Supplementary information on the data

3.1. 42 sectors of the input-output tables

Table S1. 42 sectors of the inter-provincial input-output table

	2012	2017
1	Farming, forest, livestock, and fishery products	Farming, forest, livestock, and fishery products
2	Coal mining and washing products	Coal mining and washing products
3	Crude petroleum and natural gas	Crude petroleum and natural gas
4	Metal mining products	Metal mining products
5	Non-metal and other mining products	Non-metal and other mining products
6	Food and tobacco products	Food and tobacco products
7	Textile	Textile
8	Textile apparel, shoes; leather, fur, feathers products	Textile apparel, shoes; leather, fur, feathers products
9	Furniture, timber processing products; products of wood, bamboo, cane, palm, straw	Furniture, timber processing products; products of wood, bamboo, cane, palm, straw
10	Paper, printing, and products for culture, education and sports	Paper, printing, and products for culture, education and sports
11	Refined petroleum, nuclear fuel, and coking products	Refined petroleum, nuclear fuel, and coking products
12	Chemicals	Chemicals
13	Non-metallic mineral products	Non-metallic mineral products
14	Metal smelting and rolling products	Metal smelting and rolling products
15	Metal products, except machinery and equipment	Metal products, except machinery and equipment
16	General machinery	General machinery
17	Special machinery	Special machinery
18	Transport equipment	Transport equipment
19	Electrical machinery and equipment	Electrical machinery and equipment
20	Communication equipment, computer and other electronic equipment	Communication equipment, computer and other electronic equipment
21	Instruments and meters	Instruments and meters
22	Other manufacturing	Other manufacturing; scrap and waste
23	Scrap and waste	Repair of metal products, machinery and equipment
24	Repair of metal products, machinery and equipment	Electricity and heat production and supply
25	Electricity and heat production and supply	Gas production and supply
26	Gas production and supply	Water production and supply

27	Water production and supply	Construction
28	Construction	Wholesale and retail
29	Wholesale and retail	Transport, storage and post
30	Transport, storage and post	Accommodation and catering
31	Accommodation and catering	Information transmission, software and information technology services
32	Information transmission, software and information technology services	Finance
33	Finance	Real estate
34	Real estate	Leasing and business services
35	Leasing and business services	Scientific research
36	Scientific research and technical services	Technical services
37	Water conservancy, environment and public facilities management	Water conservancy, environment and public facilities management
38	Service to households, repair and other services	Service to households, repair and other services
39	Education	Education
40	Healthcare and social work activities	Healthcare and social work activities
41	Culture, sports and entertainment	Culture, sports and entertainment
42	Public management、social security and social organizations	Public management、social security and social organizations

3.2. 31 provinces of the input-output tables

Table S2. 31 provinces of the inter-provincial input-output table

	Provinces		Provinces		Provinces
1	Beijing	12	Anhui	22	Chongqing
2	Tianjin	13	Fujian	23	Sichuan
3	Hebei	14	Jiangxi	24	Guizhou
4	Shanxi	15	Shandong	25	Yunnan
5	Inner Mongolia	16	Henan	26	Tibet
6	Liaoning	17	Hubei	27	Shaanxi
7	Jilin	18	Hunan	28	Gansu
8	Heilongjiang	19	Guangdong	29	Qinghai
9	Shanghai	20	Guangxi	30	Ningxia
10	Jiangsu	21	Hainan	31	Xinjiang
11	Zhejiang				

3.3. Leading asset managers' equity portfolio investment in China's stock market

By using the shareholding information of asset managers and the market value of public companies in China, we analyze the market value of public companies in different industries held by the top five foreign asset managers and top ten domestic asset managers. The calculation results are presented in Table S1.

Table S3. The market value held by fifteen asset managers in 2010 and 2019

a. The market value of public companies in China held by fifteen asset managers in 2010 (billion RMB)

	BlackRock	Vanguard	State Street Global	Fidelity Investments	Allianz Group	CCB Principal Asset Mgmt.	E Fund Mgmt.	Agricultural Bank of China	CITIC Securities	ICBC Credit Suisse Asset Mgmt.	China Asset Mgmt.	Harvest Fund Mgmt.	China Southern Asset Mgmt.	Bosera Asset Mgmt.	China Merchants Fund
Agriculture	51.7	9.9	5.0	0.4	0.0	40.6	98.4	4.6	5.2	45.9	305.5	270.6	144.5	27.6	56.5
Coal mining	115.7	7.5	42.5	25.0	34.4	135.6	782.5	16.7	49.3	173.4	575.4	451.8	387.5	577.0	210.9
Other mining sector	376.7	32.5	14.6	0.9	2.2	81.2	361.3	0.5	33.0	86.5	652.1	257.6	282.5	504.4	61.0
Oil processing	2.8	2.9	2.8	0.0	0.0	9.8	19.4	0.0	0.0	4.8	118.4	20.7	20.7	7.0	0.0
Chemical	176.0	91.6	120.4	578.8	18.0	290.3	1302.3	149.1	344.1	447.1	2014.9	1851.2	922.7	858.6	380.4
Non-metal products	130.8	19.5	21.1	94.5	6.8	120.3	190.8	114.2	124.2	80.1	412.7	448.7	319.8	260.5	35.8
Metal smelting and rolling	290.3	40.6	30.9	3.7	20.6	118.3	657.6	3.0	168.8	180.5	649.8	517.9	702.8	526.0	118.7
Electricity and heat	169.7	99.0	33.3	70.5	2.3	31.8	180.0	3.2	0.0	90.3	215.9	160.9	234.2	413.6	2.3
Other manufacturing sector	1353.9	420.2	312.3	683.1	98.8	589.4	3196.6	625.7	477.0	1139.4	6333.1	3047.3	3173.6	3255.4	821.6
Construction	170.8	0.6	16.3	0.0	5.5	73.6	276.7	29.4	22.6	125.7	433.4	271.8	293.1	211.6	12.6
Transportation	237.5	11.4	23.6	64.4	22.9	129.0	387.1	77.9	24.6	126.3	387.6	325.2	303.6	741.5	55.8
Finance	4670.4	0.0	137.4	0.0	57.4	612.9	2299.4	51.2	81.3	1466.7	10694.5	2130.0	1738.6	2382.3	133.3
Leasing and business services	73.6	38.8	9.3	10.2	13.9	145.6	224.5	60.5	19.9	220.4	617.4	203.9	268.0	272.5	80.4
Retail	254.0	33.6	17.6	71.8	68.6	246.0	773.4	43.3	26.8	263.5	892.6	467.5	829.5	686.1	189.3
Other service sector	348.9	101.7	137.7	134.5	22.6	359.0	1012.3	70.1	221.4	381.7	1767.2	1156.0	855.3	1229.0	326.4
Total	8422.9	909.8	924.8	1737.7	373.9	2983.5	11762.2	1249.3	1598.0	4832.3	26070.4	11581.0	10476.3	11953.1	2484.9

Data sources: S&P Capital IQ (https://www.capitaliq.com) and CSMAR (http://cndata1.csmar.com).

b. The market value of public companies in China held by fifteen asset managers in 2019 (billion RMB)

	BlackRock	Vanguard	State Street Global	Fidelity Investments	Allianz Group	CCB Principal Asset Mgmt.	E Fund Mgmt.	Agricultural Bank of China	CITIC Securities	ICBC Credit Suisse Asset Mgmt.	China Asset Mgmt.	Harvest Fund Mgmt.	China Southern Asset Mgmt.	Bosera Asset Mgmt.	China Merchants Fund
Agriculture	211.2	153.7	13.6	2.4	1.4	52.4	203.4	4.4	1.3	93.7	71.0	116.7	238.2	28.1	23.0
Coal mining	157.4	149.2	18.3	16.6	0.6	12.9	164.8	1.0	3.9	29.8	82.6	87.3	168.4	71.5	63.6
Other mining sector	231.7	188.1	16.5	6.3	0.0	28.0	178.9	16.6	12.8	106.2	415.1	272.7	259.5	466.6	20.8
Oil processing	24.1	34.9	2.1	0.0	0.0	61.0	31.3	0.1	0.1	8.2	27.4	17.6	21.0	25.3	11.6
Chemical	1592.2	1670.4	194.4	281.1	22.0	269.8	1667.1	154.2	134.8	737.4	1421.6	1141.6	1377.3	1031.1	343.2
Non-metal products	231.3	271.9	31.7	40.8	13.8	52.3	407.1	47.1	40.9	197.0	347.2	383.0	255.0	144.2	69.8
Metal smelting and rolling	352.8	311.9	20.7	3.4	0.8	39.0	371.8	22.1	20.8	84.5	464.2	281.5	319.1	388.8	31.0
Electricity and heat	305.5	221.6	56.8	47.8	6.2	50.7	387.1	2.7	10.1	103.6	418.0	216.1	364.1	328.3	83.4
Other manufacturing sector	6886.1	6899.8	623.8	1730.8	278.0	1204.9	7179.3	694.8	384.8	2313.8	6065.9	5559.9	5354.9	3093.7	1740.7
Construction	269.5	234.4	28.2	6.2	0.9	173.2	385.6	2.6	90.5	346.0	401.5	587.5	359.7	621.2	10.7
Transportation	507.9	787.6	94.0	182.5	7.6	86.5	593.8	3.4	7.5	148.3	799.0	322.7	371.3	347.8	47.0
Finance	5125.5	3238.3	456.0	6.6	62.4	296.3	3131.3	297.9	419.9	2241.0	8315.7	3059.7	3869.3	1035.3	1471.9
Leasing and business services	255.6	516.2	22.0	29.1	33.4	149.1	169.6	19.7	2.8	49.0	257.9	100.9	333.1	81.9	12.9
Retail	224.0	283.4	33.5	50.5	7.6	39.9	89.2	9.8	22.8	35.8	255.3	178.4	259.6	106.2	66.9
Other service sector	3203.1	3253.1	517.4	602.7	61.5	570.8	1832.3	356.1	169.9	869.1	1978.8	2079.8	1684.2	1277.8	324.4
Total	19577.9	18214.5	2129.1	3006.8	496.1	3086.5	16792.6	1632.4	1322.7	7363.5	21321.1	14405.5	15234.8	9047.8	4320.9

Data sources: S&P Capital IQ (https://www.capitaliq.com) and CSMAR (http://cndata1.csmar.com).

3.4. The green investment principles that asset managers follow

Table S4. The green investment principles that asset managers follow

	United Nations-supported Principles for Responsible Investment (PRI)	Climate Action 100+	The Net Zero Asset Managers Initiative	Ceres	The Institutional Investors Group on Climate Change (IIGCC)	Asia investor group on climate change (AIGCC)	Investor Group on Climate Change (IGCC)
China Asset Mgmt.	√	√					
BlackRock*	√	√	√	√	√	√	√
Vanguard*	√			√	√	√	√
China Southern Asset Mgmt.	√	√					
Bosera Asset Management	√						
E Fund Mgmt.	√	√					
Harvest Fund Mgmt.	√	√					
ICBC Credit Suisse Asset Mgmt.	√						
CCB Principal Asset Mgmt.	√						
China Merchants Fund	√						
Fidelity Investment*	√	√	√	√	√	√	√
State Street Global*	√	√	√	√	√		
CITIC Securities							
Agricultural Bank of China							
Allianz Group*	√	√	√		√	√	

Data Sources: United Nations-supported Principles for Responsible Investment (https://www.unpri.org/signatories/signatory-resources/signatory-directory); Climate Action 100+ (https://www.climateaction100.org/whos-involved/investors/); The Net Zero Asset Managers Initiative (https://www.netzeroassetmanagers.org/signatories/); Ceres (https://www.ceres.org/networks/ceres-investor-network); The Institutional Investors Group on Climate Change (https://www.iigcc.org/about-us/our-members/); Asia investor group on climate change (https://www.aigcc.net/our-members/); Investor Group on Climate Change (https://igcc.org.au/our-members/).

Note: Companies labeled with superscript "*" are foreign asset managers.

Supplementary References:

1. Microsoft. *Annual Report 2020*. <https://www.microsoft.com/investor/reports/ar20/index.html> (2021).

2. Ministry of Ecology and Environment (MEE). *Guidelines for enterprise greenhouse gas verification (trial)*. <http://www.mee.gov.cn/xxgk2018/xxgk/xxgk06/202103/t20210329_826480.html> (2021).

3. World Economic Forum. Scaling up climate action through value chain mobilization. <https://www.weforum.org/reports/scaling-up-climate-action-through-value-chain-mobilization> (2016).

4. Steininger, K. W., Lininger, C., Meyer, L. H., Munoz, P. & Schinko, T. Multiple carbon accounting to support just and effective climate policies. *Nat. Clim. Chang.* **6**, 35–41 (2016).

5. China Petroleum & Chemical Co., L. *Sustainable Development Report 2020*. <http://www.sinopec.com/listco/Resource/Pdf/2021032840.pdf> (2021).

6. Antràs, P., Chor, D., Fally, T. & Hillberry, R. Measuring the upstreamness of production and trade flows. *Am. Econ. Rev.* **102**, 412–416 (2012).

7. Fally, T. On the fragmentation of production in the us. *University of Colorado-Boulder.* (2011).

8. Wang, Z., Wei, S.-J., Yu, X. & Zhu, K. Characterizing Global Value Chains: Production Length and Upstreamness. *Natl. Bur. Econ. Res.* (2017).
