# Supplementary material for: Value chain carbon footprints of Chinese listed companies
Source: Nat Commun. 2023 May 16;14:2794. doi: 10.1038/s41467-023-38479-5 (PMC10188601; doi:10.1038/s41467-023-38479-5)
Supplement: Supplementary file 2 — Reporting Summary [file 41467_2023_38479_MOESM2_ESM.pdf]

nature portfolio

Corresponding author(s): Dabo Guan

Last updated by author(s): 2023-4-5

Reporting Summary

Nature Portfolio wishes to improve the reproducibility of the work that we publish. This form provides structure for consistency and transparency in reporting. For further information on Nature Portfolio policies, see our Editorial Policies and the Editorial Policy Checklist.

Statistics

For all statistical analyses, confirm that the following items are present in the figure legend, table legend, main text, or Methods section.

n/a	Confirmed	
☒	☐	The exact sample size (*n*) for each experimental group/condition, given as a discrete number and unit of measurement
☒	☐	A statement on whether measurements were taken from distinct samples or whether the same sample was measured repeatedly
☒	☐	The statistical test(s) used AND whether they are one- or two-sided<br>*Only common tests should be described solely by name; describe more complex techniques in the Methods section.*
☒	☐	A description of all covariates tested
☒	☐	A description of any assumptions or corrections, such as tests of normality and adjustment for multiple comparisons
☒	☐	A full description of the statistical parameters including central tendency (e.g. means) or other basic estimates (e.g. regression coefficient) AND variation (e.g. standard deviation) or associated estimates of uncertainty (e.g. confidence intervals)
☒	☐	For null hypothesis testing, the test statistic (e.g. *F*, *t*, *r*) with confidence intervals, effect sizes, degrees of freedom and *P* value noted<br>*Give P values as exact values whenever suitable.*
☒	☐	For Bayesian analysis, information on the choice of priors and Markov chain Monte Carlo settings
☒	☐	For hierarchical and complex designs, identification of the appropriate level for tests and full reporting of outcomes
☒	☐	Estimates of effect sizes (e.g. Cohen's *d*, Pearson's *r*), indicating how they were calculated

Our web collection on statistics for biologists contains articles on many of the points above.

Software and code

Policy information about availability of computer code

Data collection	No software was used for data collection.
Data analysis	We developed scripts in Matlab for data analysis, which can be accessed through DOI: 10.6084/m9.figshare.21936938.

For manuscripts utilizing custom algorithms or software that are central to the research but not yet described in published literature, software must be made available to editors and reviewers. We strongly encourage code deposition in a community repository (e.g. GitHub). See the Nature Portfolio guidelines for submitting code & software for further information.

Data

Policy information about availability of data

All manuscripts must include a data availability statement. This statement should provide the following information, where applicable:

- Accession codes, unique identifiers, or web links for publicly available datasets
- A description of any restrictions on data availability
- For clinical datasets or third party data, please ensure that the statement adheres to our policy

The data that support the findings of this study are provided in the Supplementary Information. Other data have been deposited at Figshare (DOI: 10.6084/m9.figshare.21936938). The multiregional input-output (MRIO) table used in this study is available at DOI：10.1021/acs.est.8b03424. The annual carbon emissions data used in this study is available at the Carbon Emission Accounts and Datasets for emerging economies (CEADs) (https://www.ceads.net.cn/data/province/). The

list of Chinese listed companies is available at China Stock Market & Accounting Research Database (CSMAR) (http://cndata1.csmar.com). The operating information of Chinese listed companies is obtained from their annual reports.

Human research participants

Policy information about studies involving human research participants and Sex and Gender in Research.

Reporting on sex and gender	This study analyzed carbon footprints of companies, having no relation with sex or gender.
Population characteristics	Not applicable
Recruitment	Not applicable
Ethics oversight	Not applicable

Note that full information on the approval of the study protocol must also be provided in the manuscript.

Field-specific reporting

Please select the one below that is the best fit for your research. If you are not sure, read the appropriate sections before making your selection.

☐ Life sciences ☐ Behavioural & social sciences ☒ Ecological, evolutionary & environmental sciences

For a reference copy of the document with all sections, see nature.com/documents/nr-reporting-summary-flat.pdf

Ecological, evolutionary & environmental sciences study design

All studies must disclose on these points even when the disclosure is negative.

Study description	We trace the carbon emissions embodied in the value chains of listed companies in China.
Research sample	As public awareness of climate change has been rising over the past years, the public are paying more and more attention to climate risks associated with their investment in the stock market. The present study focuses on all Chinese listed companies over the period 2010-2019 whose shares can be traded by the public on a country's main stock market.
Sampling strategy	All Chinese listed companies are included in our analysis, no sampling strategy is used.
Data collection	The national multi-regional input-output (MRIO) table is obtained from the National Bureau of Statistics of China. Data on the annual carbon emissions are collected from the Carbon Emission Accounts and Datasets for emerging economies(CEADs). The operating information of listed companies is collected manually from their annual reports. The data is recorded by Zengkai Zhang and Jiaoyan Li.
Timing and spatial scale	We began collecting the data from January, 2021. We stop data collecting in July, 2022.
Data exclusions	No data were excluded.
Reproducibility	Authors repeat the calculation process over ten times and all attempts at replication were successful.
Randomization	We did not allocate listed companies into groups by their industry attribute.
Blinding	In our non-experimental design, we use operating data of Chinese listed companies collected from their annual reports, which had been published to the public, to estimate their carbon footprints. Blinding is hence not necessary.

Did the study involve field work? ☐ Yes ☒ No

Reporting for specific materials, systems and methods

We require information from authors about some types of materials, experimental systems and methods used in many studies. Here, indicate whether each material, system or method listed is relevant to your study. If you are not sure if a list item applies to your research, read the appropriate section before selecting a response.

Materials & experimental systems

n/a	Involved in the study
☒	☐ Antibodies
☒	☐ Eukaryotic cell lines
☒	☐ Palaeontology and archaeology
☒	☐ Animals and other organisms
☒	☐ Clinical data
☒	☐ Dual use research of concern

Methods

n/a	Involved in the study
☒	☐ ChIP-seq
☒	☐ Flow cytometry
☒	☐ MRI-based neuroimaging
